# Preoperative Ballon Aortic Valvuloplasty vs. Conservative Management in Patients Undergoing Hip Fracture Surgery with Underlying Severe Aortic Stenosis

**DOI:** 10.3390/jcm15208160

**Published:** 2026-10-20

**Authors:** Antonio De Vita, Marcello Covino, Francesco Mollo, Matteo Vumbaca, Chiara Napolitano, Saverio Tremamunno, Nello Cambise, Rosaria Natali, Marco Rossi, Tiziana Bove, Raffaele Vitiello, Giulio Maccauro, Francesco Franceschi, Carlo Trani, Francesco Burzotta, Gaetano Antonio Lanza

**Affiliations:** 1 Faculty of Medicine and Surgery, Università Cattolica del Sacro Cuore, Rome Campus, 00168 Rome, Italy; macovino@gmail.com (M.C.); francescomollo989@gmail.com (F.M.); gaetanoantonio.lanza@unicatt.it (G.A.L.); 2 Department of Cardiovascular Sciences, Fondazione Policlinico Universitario a. Gemelli IRCCS, 00168 Rome, Italy; 3 Department of Emergency Medicine, Fondazione Policlinico Universitario a. Gemelli IRCCS, 00168 Rome, Italy; 4 Department of Emergency, Anesthesiologic and Reanimation Sciences, Fondazione Policlinico Universitario a. Gemelli IRCCS, 00168 Rome, Italy; 5 Department of Ageing, Neurosciences, Head-Neck and Orthopedics Sciences, Fondazione Policlinico Universitario a. Gemelli IRCCS, 00168 Rome, Italy

**Keywords:** balloon aortic valvuloplasty, severe aortic stenosis, hip fracture, preoperative management, mortality

## Abstract

**Background:** Old adults with hip fracture (HFx) and concomitant severe aortic stenosis (AS) represent a high-risk population in whom urgent orthopedic repair is usually required, while definitive valve replacement is rarely feasible preoperatively. The clinical benefit of balloon aortic valvuloplasty (BAV) as a bridging strategy remains uncertain. **Methods:** In this observational study, we included patients admitted to our Emergency Department for HFx who had concomitant severe AS. Management of severe AS consisted of either conservative therapy or preoperative BAV. The early clinical endpoints included 30-day mortality, time to surgery, length of intensive care unit and hospital stay and need for mechanical ventilation. The late clinical endpoint was 180-day all-cause mortality. **Results:** Among 3958 patients admitted for HFx fracture, 46 (1.2%) were found to have severe AS and fulfilled the inclusion criteria. Of these, 12 patients (26.1%) underwent preoperative BAV and 34 (73.9%) received conservative management. Thirty-day mortality was 8.3% in the BAV group and 23.5% in the conservative group (HR 0.32; 95%CI 0.04–2.60; *p* = 0.29). Time to surgery was longer in the BAV group (*p* < 0.001) with concomitantly longer hospital (*p* = 0.001) and ICU (*p* = 0.041) stays. Preoperative BAV was independently associated with lower 180-day mortality (HR 0.13; 95%CI 0.03–0.63; *p* = 0.011). **Conclusions**: In patients with severe AS undergoing urgent HFx repair, preoperative BAV was associated with reduced 180-day mortality, suggesting a potential long-term prognostic benefit, which warrants confirmation in larger prospective studies. However, it was not associated with significantly better 30-day survival and was accompanied by surgical delay and greater healthcare resource utilization.

## 1. Introduction

Aortic stenosis (AS) is one of the most prevalent valvular heart diseases in Europe and North America [1]. Its prevalence increases with rising life expectancy, affecting up to 10% of individuals aged over 80 years [2,3].

In patients undergoing non-cardiac surgery, severe symptomatic AS is associated with an increased risk of death and major adverse cardiovascular events (MACE) in the perioperative period [4]. Accordingly, guidelines recommend deferring elective non-cardiac surgery to after valve replacement, whenever possible [5,6].

In patients requiring urgent surgical intervention, however, the management of severe AS remains poorly defined. While a definitive treatment of the valvular disease through surgical or transcatheter aortic valve replacement (SAVR or TAVR) cannot be performed in this setting, the potential benefit of increasing the aortic valve area by balloon aortic valvuloplasty (BAV) on clinical outcomes has not been adequately investigated [7,8].

Although the clinical scenario of severe AS in the context of urgent non-cardiac surgery is uncommon across most types of interventions, it may affect a considerable proportion, up to 5–10% of patients admitted to the Emergency Department (ED) for hip fracture (HFx), since this represents a rather frequent urgent non-cardiac intervention in the elderly population [9]. For instance, the presence of severe AS in HFx patients has been associated with a significant increase in the risk of perioperative cardiac complications, with a 3- to 4-fold higher 30-day mortality rate (between 7 and 14%) and a 1-year mortality rate reaching 50% [9,10,11,12,13]. Whether BAV may reduce this risk without affecting delay in HFx surgery and functional recovery remains unclear.

Thus, the aim of this study was to evaluate whether preoperative BAV, compared with conservative management, may impact early and medium-term postoperative outcomes in AS patients undergoing surgery for HFx.

## 2. Material and Methods

### 2.1. Study Population

We conducted a single-center prospective observational study, including all patients admitted to the ED of our Tertiary University Hospital between 1 September 2022 and 31 December 2024, with a diagnosis of HFx requiring orthopedic surgery and echocardiographic evidence of severe AS. In January 2024, due to a lower-than-anticipated number of prospectively enrolled cases, a post-hoc decision was made to expand the study population by including an additional retrospective cohort of patients, whose clinical data had been previously collected and stored in our institutional database between January 2015 and August 2022. Retrospective cases were included only if they fulfilled the same predefined eligibility criteria applied to prospectively enrolled participants. Accordingly, the study adopted an ambispective cohort design, combining both prospectively enrolled and retrospectively identified patients, all assessed with the same methodological framework and standardized data collection procedures.

Severe AS was diagnosed using current transthoracic echocardiography (TTE) criteria: aortic valve area (AVA) ≤ 1.0 cm^2^ and/or a mean transvalvular gradient ≥ 40 mmHg and/or peak aortic Doppler jet velocity ≥ 4.0 m/s [14,15].

Patients were excluded when one or more of the following conditions were present: (1) history of aortic valve replacement or repair; (2) hemodynamical instability at hospital admission; (3) high gradients or velocities attributable to increased cardiac output (anaemia, septic shock, etc.); (4) concomitant diseases that have influence on Doppler indices of AS (hypertrophic obstructive cardiomyopathy, subvalvular or supravalvular aortic stenosis, coarctation of the aorta or complex congenital heart diseases); and (5) incomplete clinical documentation.

Orthopedic surgical management included closed reduction with percutaneous fixation, intramedullary nailing, arthroplasty (hemiarthroplasty or total hip arthroplasty) and open reduction with internal fixation (ORIF). The choice of the procedure was determined by the treating orthopedic surgery team (based on fracture pattern, patient comorbidity burden, baseline functional status, clinical presentation and surgical expertise). Management of severe AS consisted of either conservative therapy or preoperative BAV. In the prospective cohort, treatment allocation was determined by a multidisciplinary Heart Team, which integrated comorbidity burden, frailty status, echocardiographic severity of stenosis, anticipated intolerance to anesthesia, presence of severe valve-related symptoms, overall perioperative risk as assessed by the ASA score, as well as fracture pattern and clinical presentation.

All patients gave written informed consent to the treatment and collection of clinical data for research purposes at the time of admission according to the institutional protocol. The study was approved by the Institutional Review Board of Fondazione Policlinico Universitario “A. Gemelli” IRCCS-Rome (#0025817/22; Study ID: #5121) and conducted in accordance with the Declaration of Helsinki.

### 2.2. Data Collection

Data were recovered from prospectively collected electronic medical records of our institutional database. For each patient, the following data were collected: demographic characteristics, comorbidities as assessed by the American Society of Anesthesiologists (ASA) classification and Charlson Comorbidity Index, symptoms potentially related to AS, echocardiographic parameters, time from admission to surgery, type of HFx surgery, type of anesthesia and strategy for aortic stenosis management.

Baseline clinical comorbidities, including hypertension, diabetes mellitus, history of heart failure, known coronary artery disease (CAD), atrial fibrillation, chronic obstructive pulmonary disease (COPD), chronic kidney disease, prior cerebrovascular events and cognitive impairment, were identified from the medical history recorded in the ED reports. These conditions were considered present only when documented as pre-existing diagnoses and were not newly diagnosed as part of the study.

Vital signs at ED admission were recorded, including blood pressure (BP), heart rate (HR), respiratory rate, temperature and peripheral oxygen saturation. In addition, laboratory tests were also collected, including serum creatinine, haemoglobin, platelet count, white blood cell count, fibrinogen, glucose, high-sensitivity troponin I, N-Terminal Pro-B-Type Natriuretic Peptide (NT-proBNP) and C-reactive protein (CRP).

All echocardiograms were performed in accordance with current European and American Society of Echocardiography recommendations [3,14,15]. In patients with multiple echocardiograms, the exam closest to the time of surgery was considered. Aortic valve parameters (AVA and AVA index, peak aortic jet velocity and mean aortic valve gradient), as well as left ventricular size, ejection fraction, left ventricular diastolic function and estimated systolic pulmonary artery pressure (SPAP) (calculated adding right ventricle/right atrium pressure gradient to the estimated right atrial pressure assessed by inferior vena cava diameter and response to respiratory acts) were extracted from the echocardiography database.

### 2.3. Study Endpoints

The study endpoints were categorized according to the early and late postoperative clinical course. Early clinical course endpoints included 30-day all-cause mortality, time to surgery, length of hospital staying, duration of intensive care unit (ICU) admission and the need for mechanical ventilation support. Survival at 180 days postoperatively represents the late-course endpoint of the study. Vital status at 30 and 180 days was primarily determined through consultation of the Health System database of Regione Lazio, Italy; when this was not feasible, vital status was ascertained through telephone interviews of patients or relatives.

### 2.4. Statistical Analysis

Continuous variables are reported as means with standard deviations when approximately normally distributed and as median with interquartile range when non-normally distributed. Categorical variables are reported as numbers and proportions. The distribution of continuous variables was assessed using the Shapiro–Wilk test. Patients were divided into two groups according to the management strategy (BAV vs. conservative strategy). The comparisons between groups of continuous variables were done by independent *t*-test or Mann–Whitney test, as indicated, whereas proportions were compared by Fisher exact test. Within-group comparisons were done by paired samples *t*-test or Wilcoxon signed-rank test, as appropriate.

Associations with 30-day and 180-day all-cause mortality were assessed using Cox proportional hazards regression models. Given the limited sample size and number of outcome events, the multivariable analyses were considered exploratory and hypothesis-generating. Variables with *p* < 0.10 in univariable analyses were considered as candidate covariates. To limit model overfitting, the final models were restricted to a small number of variables selected according to clinical relevance. Results are reported as hazard ratios with 95% confidence intervals. All tests were two-sided and *p* < 0.05 was considered statistically significant.

Data were analyzed by the SPSS 21.0 statistical software (SPSS Italia, Inc., Florence, Italy).

## 3. Results

### 3.1. General Results

Overall, 3958 patients were admitted to the ED of our Hospital for HFx fracture during the entire study period. Among these, 195 patients were found to have concomitant AS at cardiological evaluation. Eighty-nine patients (45.6%) had mild AS, 58 (29.7%) moderate AS and 48 (24.6%) severe AS (1.2% of the whole population of HFx patients). Two patients with severe AS were excluded due to cardiogenic shock at presentation, requiring inotropic and vasopressor support (Figure 1).

Among the 46 patients with severe AS who fulfilled the inclusion criteria, 12 patients were prospectively enrolled and 34 were retrospectively identified and included. Overall, 12 patients (26.1%) underwent preoperative BAV, whereas 34 patients (73.9%) received conservative treatment. All enrolled patients underwent HFx surgery.

The main demographic and clinical characteristics and laboratory findings of the two groups are summarized in Table 1. Patients managed conservatively were older than those treated with preoperative BAV (88.6 ± 5.4 vs. 83.7 ± 7.4 years, *p* = 0.020), but no other differences were observed between the two groups in terms of comorbidities, cardiovascular risk factors, laboratory parameters or pharmacological therapy.

At echocardiography, there were no significant differences between the two groups in mean aortic gradient (49.0 ± 16.6 mmHg vs. 42.0 ± 22.0 mmHg; *p* = 0.40), peak aortic velocity (4.2 ± 0.6 vs. 4.1 ± 0.4 m/s; *p* = 0.52), LVEF (48.1 ± 15.0 vs. 52.3 ± 13.0%, *p* = 0.36) and systolic pulmonary artery pressure (SPAP) (39.5 ± 13.9 vs. 49.0 ± 15.5 mmHg; *p* = 0.14). Conversely, patients undergoing BAV had a significant larger end-diastolic volume (114.5 ± 55.5 mL vs. 77.0 ± 30.6 mL; *p* = 0.032) and exhibited a trend toward a smaller AVA (0.59 ± 0.17 cm^2^ vs. 0.72 ± 0.19 cm^2^; *p* = 0.082) compared with those managed conservatively (Table 2).

### 3.2. Early Clinical Course

There were no significant differences between the two groups as far as the type of HFx surgery and anesthetic approach were concerned. By contrast, time from ED admission to surgery was significantly longer in the BAV group (280.1 ± 169 vs. 54.6 ± 42 h, *p* < 0.001). HFx surgery was performed within 48 h of ED admission in 23 patients (67.6%) in the conservatively managed group, whereas no patients in the BAV group underwent surgery within the same timeframe (*p* < 0.001). Moreover, both length of hospital stay (25.7 ± 19.1 vs. 11.9 ± 7.2 days, *p* = 0.001) and duration of ICU admission (50.6 ± 53.6 vs. 25.5 ± 19.8 h, *p* = 0.041) were significantly longer in the BAV group. However, conservatively managed patients showed a trend toward increased requirement for postoperative mechanical ventilation (47.1% vs. 16.7%, *p* = 0.09) (Figure 2).

Among patients who underwent BAV, post-procedural echocardiographic assessment showed a reduction in peak aortic velocity from 4.2 ± 0.6 to 3.3 ± 0.5 m/s (*p* < 0.001) and in mean aortic gradient from 49.0 ± 16.6 to 30.5 ± 8.8 mmHg (*p* < 0.001). AVA increased from 0.59 ± 0.20 to 1.05 ± 0.30 cm^2^ (*p* < 0.001) and a modest increase in LVEF was also observed (from 48.1 ± 15.0% to 50.2 ± 13.4%, *p* = 0.047). No significant changes were observed in left ventricular end-diastolic diameter, end-diastolic volume or SPAP (Table 3).

The rate of all-cause mortality at 30 days was 8.3% in the BAV group and 23.5% in the conservative group (unadjusted hazard ratio (HR) 0.32, 95% CI: 0.04–2.60, *p* = 0.29). (Figure 3). The association between clinical variables and 180-day all-cause mortality is summarized in Table 4. In the univariable Cox regression analyses, previous TIA/stroke was associated with 30-day all-cause mortality (*p* = 0.018), whereas male sex (*p* = 0.090) and atrial fibrillation (*p* = 0.055) showed a trend. In the multivariable Cox regression analysis, prior cerebrovascular disease remained associated with 30-day all-cause mortality (HR 4.84; 95% CI 1.12–20.8; *p* = 0.034).

### 3.3. Late Clinical Course

The rate of all-cause mortality at 180 days was 16.7% in the BAV group and 52.9% in the conservative group (unadjusted HR 0.25; 95% CI: 0.06–1.08; *p* = 0.063). Furthermore, four patients (8.7%) underwent TAVR within 180 days following HFx surgery, 3/12 (25.0%) in the BAV group and 1/34 (2.9%) in the conservative group (*p* = 0.049).

The association between clinical variables and 180-day all-cause mortality is summarized in Table 5. In the overall cohort, unadjusted analysis showed that previous TIA/stroke (*p* = 0.021) and dyslipidaemia (*p* = 0.038) were associated with 180-day all-cause mortality, whereas male sex (*p* = 0.067), pre-operative BAV (*p* = 0.063) and higher white blood cell count (*p* = 0.062) showed a trend.

In the multivariable Cox regression analysis, preoperative BAV was associated with a lower risk of 180-day all-cause mortality compared with conservative management (HR 0.13; 95% CI 0.03–0.63; *p* = 0.011). Prior cerebrovascular disease (HR 9.10; 95% CI, 2.27–36.4; *p* = 0.002) and male sex HR 2.75; 95% CI, 1.04–7.27; *p* = 0.041) were also associated with 180-day mortality (Figure 4).

## 4. Discussion

The management of patients with severe AS who develop HFx requiring urgent surgical intervention is highly challenging, particularly owing to the absence of evidence derived from randomized clinical trials. In this setting, BAV, due to its relatively low invasiveness and easier procedural feasibility, may represent a more appropriate bridging strategy compared with SAVR or TAVR, with the aim of reducing the risks associated with emergency HFx surgery.

In this observational single-centre study, the use of BAV was associated with: (1) increased complexity in clinical management, as reflected by delays in definitive surgical treatment and prolonged intensive care unit staying and (2) a potentially beneficial effect on patient outcomes, as suggested by a strong signal toward improved 180-day survival.

Our data failed to demonstrate that a preoperative BAV strategy, as compared with a conservative approach, translates into a statistically significant lower perioperative 30-day mortality. Despite the BAV group showing a lower mortality compared to the conservative group (8.3% vs. 23.5%), indeed, the difference did not achieve statistical significance, whereas it showed a significantly more prolonged time to surgery (about 280 vs. 55 h) and longer ICU and overall hospital stay.

The lack of statistically significant difference in 30-day mortality between the two groups, however, might be related to the low number of patients undergoing BAV in our cohort, thus suggesting that the possible protective role of pre-operative BAV in these patients may deserve evaluation in larger studies.

Within the limitations of an observational single-centre study, the present findings, specifically a significant association between preoperative BAV and all-cause mortality at 180 days, provide suggestions for the use of preoperative BAV in patients with AS requiring urgent HFx surgical repair. Concomitantly, they highlight the need for improved management strategies in this population, with particular emphasis on optimizing the timing of orthopaedic intervention to facilitate postoperative recovery and early mobilization, while enhancing the efficiency of healthcare resource utilization [16,17].

To the best of our knowledge, there are currently no randomized controlled trials comparing operative strategies for patients with severe AS requiring urgent non-cardiac surgery such as HFx repair. Contemporary series of HFx patients report 30-day mortality around 5–10% and 180-day mortality between 12% and 20% [13,18,19,20,21,22]. However, evidence regarding the influence of AS on peri-operative outcome is conflicting, with some studies showing no difference in 30-day mortality after surgical HFx repair between patients with asymptomatic severe AS and controls [23], but others showing higher mortality rates up to one year in patients with AS [9,10,11,12].

Our findings are consistent with those reported by Ferré et al. [18], who, in a small retrospective cohort of patients with HFx and severe AS, demonstrated a significant reduction in overall mortality with preoperative BAV, albeit at the expense of a longer time to surgery.

Notably, in our cohort, only a limited number (four) of patients underwent definitive TAVR within the 6-month follow-up period after HFx surgery, with a higher proportion in the BAV group than in the conservative-treatment group. The measured comorbidity burden, including the Charlson Comorbidity Index, did not differ significantly between the two groups, although conservatively managed patients were older. However, reasons for proceeding with or withholding TAVR were not prospectively collected. Therefore, the observed difference may reflect several factors, including age, unmeasured frailty or functional status, post-fracture recovery, patient or family preference, anatomical or procedural eligibility, survival to clinical reassessment and clinician selection. The higher rate of subsequent TAVR in the BAV group may reflect differences in patient selection and recovery rather than the effect of the initial treatment strategy. Particularly, patients had to survive and recover sufficiently after hip fracture surgery to be considered for TAVR. Consequently, we cannot determine whether subsequent TAVR influenced 180-day mortality or simply identified patients with a more favourable clinical profile.

It is important to highlight that the variables associated with mortality observed in our cohort, including prior cerebrovascular disease, atrial fibrillation and male sex, are concordant with evidence from previous larger studies [4,13,22]. These variables likely represent surrogates for systemic vulnerability, encompassing frailty, heightened thromboembolic risk and the burden of coexisting conditions, thereby underscoring the complex interplay of factors that drive mortality in elderly patients with cardiovascular disease.

Within this context, the perioperative course also underscores a relevant practical trade-off. BAV recipients experienced greater resource utilization (longer ICU and hospital stays) and substantial delays to fracture repair, likely reflecting procedural logistics and post-BAV monitoring. Indeed, the longer ICU stay observed in the BAV group may reflect the need for peri-procedural monitoring and the greater complexity of the clinical pathway associated with the additional intervention. Conversely, the greater postoperative mechanical ventilation burden observed in the conservative group may reflect cardiopulmonary decompensation in a subset of patients. However, ICU length of stay was assessed across the entire population, whereas the duration of mechanical ventilation was assessed only in the subset of patients who required postoperative respiratory support. Therefore, given the small number of ventilated patients and the absence of standardized criteria for initiating and discontinuing mechanical ventilation, these findings should be considered descriptive and do not allow a mechanistic or causal interpretation.

Taken together our data may support a pragmatic approach: prioritize expedited HFx surgery with meticulous hemodynamic management for most patients with severe AS, reserve BAV for a narrow subset with refractory decompensation or anaesthetic intolerance and then pursue timely definitive SAVR or TAVR once orthopaedic stabilization is achieved.

### Study Limitations

Our study has some limitations that should be acknowledged. First, the retrospective non-randomized nature of the study does allow to exclude the influence of undetermined variables on the results.

Second, the small sample size of patients is likely associated with a reduced statistical power and limits the generalizability of the data. Moreover, the post-hoc decision to expand the study population by including a retrospectively identified cohort may have introduced selection bias, temporal heterogeneity in clinical management and differences in data completeness, despite the application of the same eligibility criteria and variable definitions to both cohorts.

Third, the multivariable analyses should be considered as exploratory due to the small sample size and number of outcome events, particularly for 30-day mortality, which may result in overfitting and imprecise estimates, as suggested by the wide confidence intervals. Accordingly, the adjusted associations should not be interpreted as causal or as validated prognostic effects and require confirmation in larger independent cohorts.

Fourth, the indication to BAV or conservative therapy was carefully assessed by a heart team in individual patients; thus, the results may have been influenced by optimal selection of patients to address interventional or conservative management. Furthermore, standardized assessments of cognitive function, functional status, frailty and nutritional status, including the Mini Mental State Examination (MMSE), Barthel Index and serum albumin levels, were not systematically available across the entire cohort. Therefore, residual differences in these clinically relevant domains may have influenced treatment allocation and outcomes.

Fifth, a late echocardiographic follow-up was not performed at a uniform time point and was not available for the entire BAV group. The long-term effects of BAV on AS and their relationship with clinical outcomes were therefore not assessed in our study, while they might have given more information about the implications of BAV treatment.

Finally, the limited number of patients who underwent SAVR or TAVR at 180-day follow-up constrains inference about optimal sequencing and timing of definitive valve therapy post-HFx and likely amplifies the observed late-mortality differences.

## 5. Conclusions

In older adults with severe AS undergoing urgent HFx repair, preoperative BAV was not associated with a statistically significant improvement in 30-day survival and was accompanied by delays to surgery as well as prolonged ICU and hospital stays. Conversely, preoperative BAV was associated with a significant improvement in 180-day all-cause mortality compared with conservative management, highlighting a potential mid-term survival advantage that deserves further assessment in larger, adequately powered studies.

## Figures and Tables

**Figure 1:**
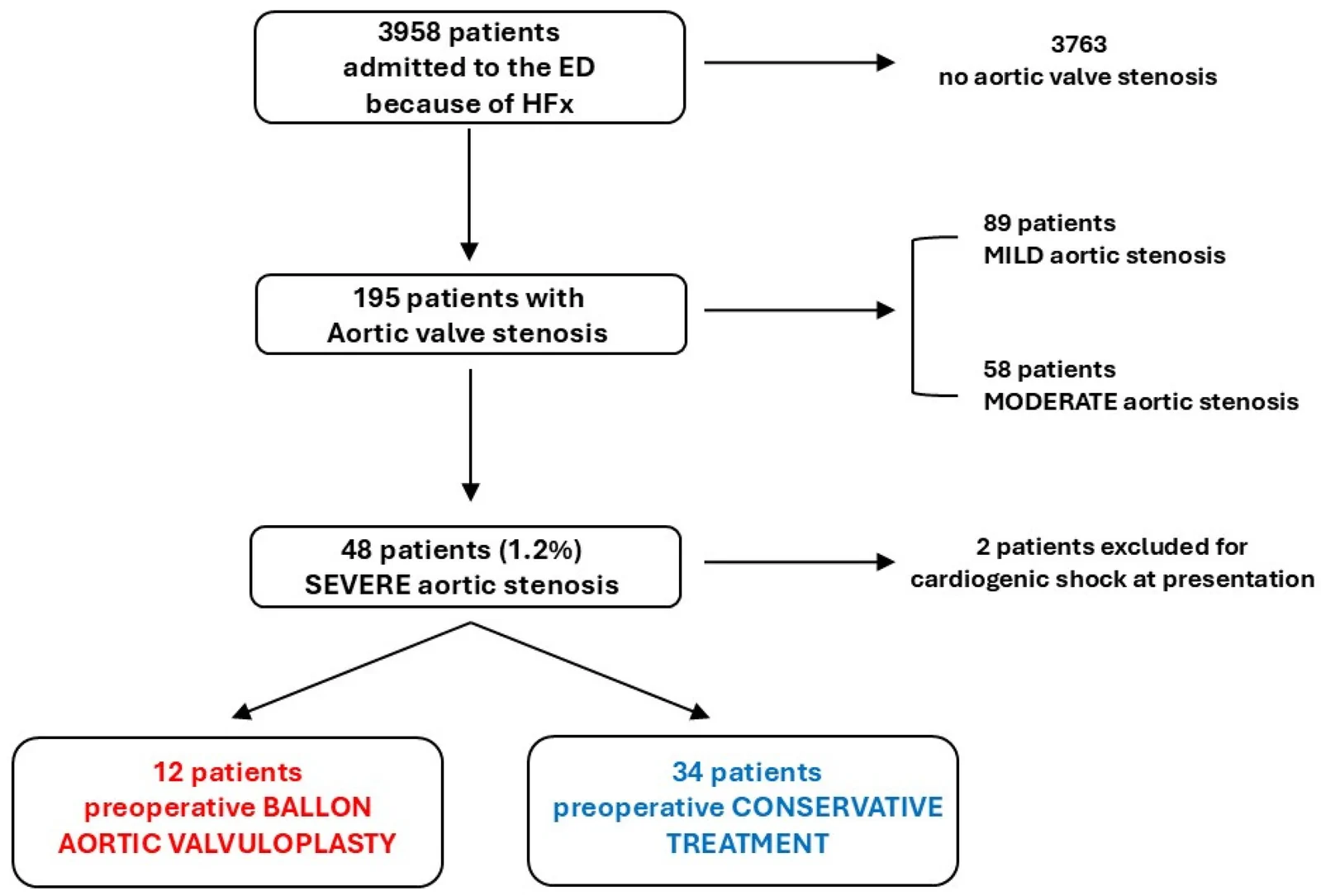
Flow chart of patient enrollment. ED = emergency department; HFx = hip fracture.

**Figure 2:**
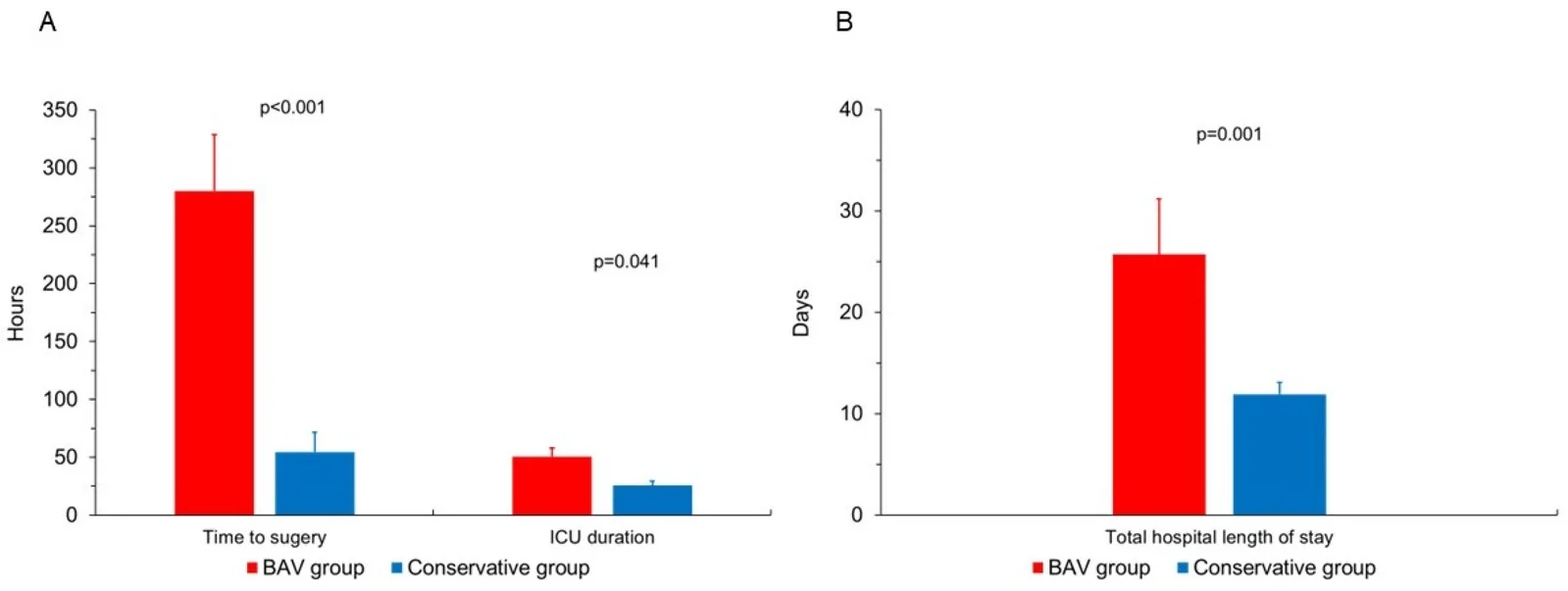
Comparison of time to surgery and intensive care unit (ICU) duration (hours) between patients undergoing preoperative balloon aortic valvuloplasty (red columns) and those managed conservatively (blue columns) (**A**), and comparison of total hospital length of stay (days) between the two groups (**B**).

**Figure 3:**
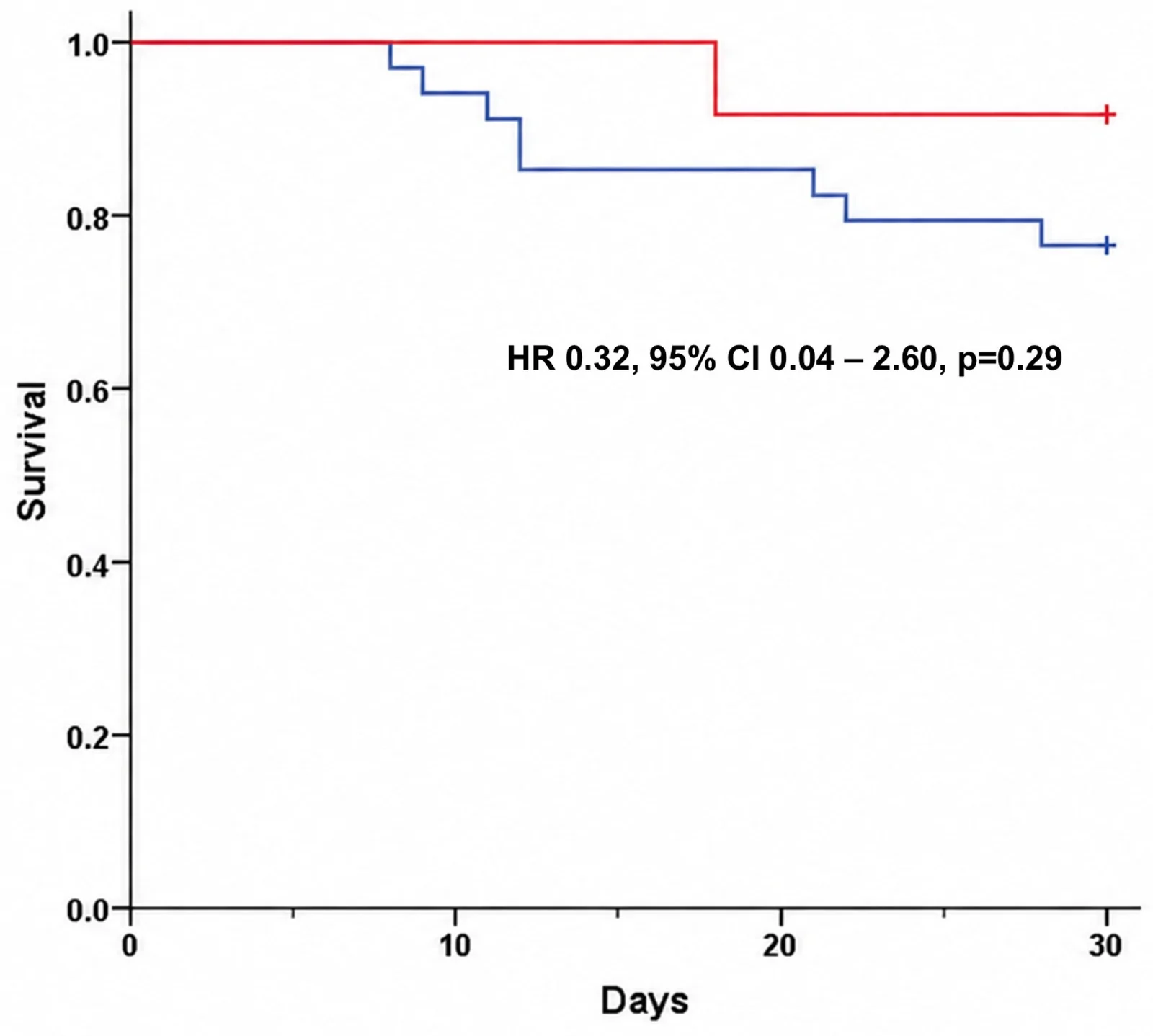
Kaplan–Meier estimates of 30-day all-cause mortality, comparing patients undergoing preoperative balloon aortic valvuloplasty (BAV group; red line) with those managed conservatively (conservative group; blue line).

**Figure 4:**
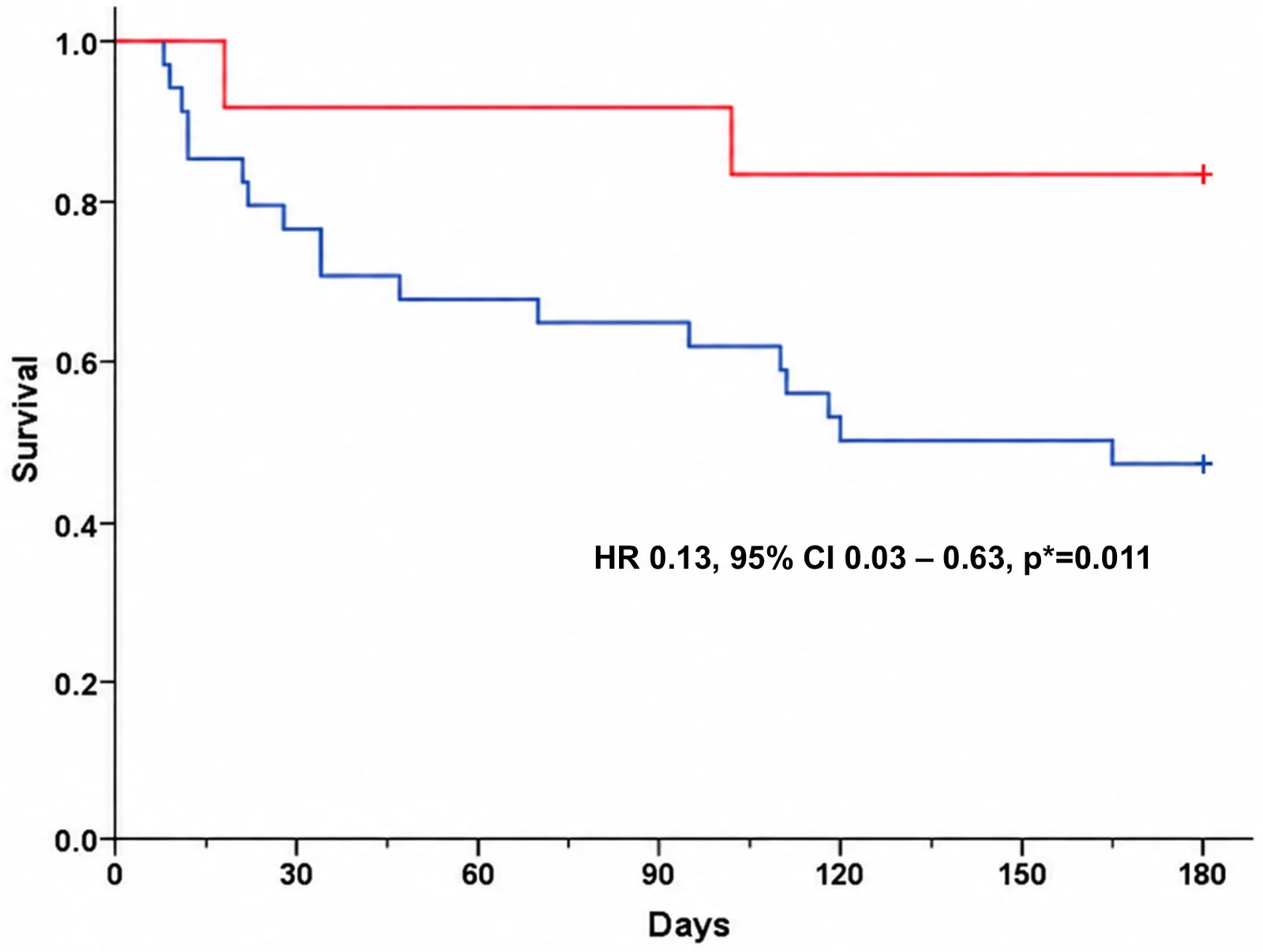
Kaplan–Meier estimates of 180-day all-cause mortality, comparing patients undergoing preoperative balloon aortic valvuloplasty (BAV group; red line) with those managed conservatively (conservative group; blue line). * *p*-value derived from the multivariable Cox regression analysis.

**Table 1:** Main clinical characteristics e laboratory findings at Emergency Department admission of the two groups of patients.

	BAV Group (*n* = 12)	Conservative Group (*n* = 34)	*p*
Age (years)	83.7 ± 7.4	88.6 ± 5.4	**0.020**
Male sex (%)	3 (25.0)	8 (23.5)	1.00
Hypertension (%)	12 (100)	30 (88.2)	0.56
Diabetes mellitus (%)	5 (41.7)	5 (14.7)	0.098
Dyslipidemia (%)	9 (75.0)	13 (38.2)	**0.044**
Heart failure (%)	7 (58.3)	25 (73.5)	0.47
Known CAD (%)	6 (50.0)	10 (29.4)	0.29
Atrial fibrillation (%)	1 (8.3)	12 (35.3)	0.13
Previous TIA/stroke (%)	2 (16.7)	3 (8.8)	0.59
Cronic Kidney disease (%)	3 (25.0)	10 (29.4)	1.00
COPD (%)	2 (16.7)	12 (35.3)	0.29
Preexisting cognitive impairment (%)	2 (16.7)	5 (14.7)	1.00
Charlson comorbidity index	6.1 ± 1.3	5.7 ± 2.2	0.58
*Laboratory findings*			
Haemoglobin (g/dL)	11.4 ± 1.5	10.7 ± 1.7	0.24
Creatinine (mg/dL)	1.06 ± 0.4	1.39 ± 1.3	0.40
Serum glucose (mg/dL)	136.2 ± 76.8	132.2 ± 43.9	0.82
Fibrinogen (mg/dL)	406.2 ± 108.1	440.8 ± 145.7	0.46
White blood cells (×10^9^/L)	11.5 ± 9.9	11.1 ± 3.8	0.84
Platelets (×10^9^/L)	222.8 ± 64.1	239.4 ± 71.9	0.48
hs-cTnI (ng/L) *	76.0 (42.5–255)	47.0 (21–131)	0.51
NT-proBNP (pg/mL) *	18,561 (2712–33,220)	4550 (2616–16,708)	0.33
C-reactive-protein (mg/L) *	23.7 (1.8–101.9)	45.1 (18.9–94.3)	0.57
Heart rate (bpm)	78.8 ± 15	85.4 ± 15	0.19
Systolic blood pressure (mmHg)	129.3 ± 21	133.1 ± 27	0.66
Diastolic blood pressure (mmHg)	74.1 ± 10	74.3 ± 16	0.97
SpO_2_ (%)	94.4 ± 5	95.1 ± 4	0.61

CAD = coronary artery disease; TIA = transient ischemic attack; COPD = chronic obstructive pulmonary disease; hs-cTnI = high sensitivity-cardiac troponin I; NT-proBNP = N-Terminal Pro-B-Type Natriuretic Peptide; and SpO_2_ = peripheral capillary oxygen saturation. * Median (interquartile range). Bold results are statistically significant.

**Table 2:** Main pre-operative echocardiographic findings of the two groups of patients.

	BAV Group (*n* = 12)	Conservative Group (*n* = 34)	*p*
End diastolic diameter (mm)	49.4 ± 8.5	43.1 ± 8.7	0.18
End diastolic volume (mL)	114.5 ± 55.5	77.0 ± 30.6	**0.032**
LVEF (%)	48.1 ± 15.0	52.3 ± 13.0	0.36
Peak aortic velocity (m/s)	4.2 ± 0.6	4.1 ± 0.4	0.52
Mean aortic gradient (mmHg)	49.0 ± 16.6	42.0 ± 22.0	0.40
AVA-Doppler (cm^2^)	0.59 ± 0.2	0.72 ± 0.19	**0.082**
DVI	0.20 ± 0.05	0.23 ± 0.06	0.20
SPAP (mmHg)	39.5 ± 13.9	49.0 ± 15.5	0.14

LVEF = left ventricular ejection fraction; AVA = aortic valve area; DVI = Doppler velocity index; and SPAP = systolic pulmonary artery pressure. Bold results are statistically significant.

**Table 3:** Pre- and post-balloon aortic valvuloplasty echocardiographic findings in patients undergoing the procedure.

	Pre BAV	Post BAV	*p*
End diastolic diameter (mm)	49.4 ± 8.5	49.5 ± 4.1	0.11
End diastolic volume (mL)	114.5 ± 55.5	107.6 ± 50.3	0.25
LVEF (%)	48.1 ± 15.0	50.2 ± 13.4	**0.047**
Peak aortic velocity (m/s)	4.2 ± 0.6	3.3 ± 0.5	**<0.001**
Mean aortic gradient (mmHg)	49.0 ± 16.6	30.5 ± 8.8	**<0.001**
AVA-Doppler (cm^2^)	0.59 ± 0.2	1.05 ± 0.3	**<0.001**
DVI	0.20 ± 0.05	0.28 ± 0.05	**0.024**
SPAP (mmHg)	39.5 ± 13.9	42.1 ± 22.9	0.62

BAV = balloon aortic valvuloplasty; LVEF = left ventricular ejection fraction; AVA = aortic valve area; DVI = Doppler velocity index; and SPAP = systolic pulmonary artery pressure. Bold results are statistically significant.

**Table 4:** Univariable and multivariable analyses evaluating all-cause mortality at 30-day follow-up.

	DEAD (*n* = 9)	ALIVE (*n* = 37)	*p* Univariable	HR	95% C.I.	*p* Multivariable
Pre-operative BAV (%)	1 (11.1)	11 (29.7)	0.29			
Age (years)	87.6 ± 4.9	87.3 ± 6.7	0.90			
Male sex (%)	4 (44.4)	7 (18.9)	0.090			
Hypertension (%)	9 (100.0)	37 (100.0)	1.00			
Diabetes mellitus (%)	0 (0)	10 (27.0)	0.30			
Dyslipidaemia (%)	3 (33.3)	19 (51.4)	0.33			
Heart failure (%)	7 (77.8)	25 (67.6)	0.51			
Known CAD (%)	4 (44.4)	12 (32.4)	0.56			
Atrial fibrillation (%)	5 (55.6)	8 (21.6)	0.055			
Previous TIA/stroke (%)	3 (33.3)	2 (5.4)	0.018	**4.84**	**1.12–20.8**	**0.034**
Chronic Kidney disease (%)	1 (11.1)	12 (32.4)	0.23			
COPD (%)	4 (44.4)	10 (27.0)	0.28			
Preexisting cognitive impairment (%)	1 (11.1)	6 (16.2)	0.66			
Charlson comorbidity index	5.9 ± 1.4	5.8 ± 2.1	0.91			
*Laboratory findings*						
Haemoglobin (g/dL)	10.4 ± 2.1	11.0 ± 1.5	0.44			
Creatinine (mg/dL)	1.0 ± 0.3	1.4 ± 1.3	0.41			
Serum glucose (mg/dL)	113 ± 27	135 ± 57	0.19			
Fibrinogen (mg/dL)	402 ± 103	439 ± 144	0.49			
White blood cells (×10^9^/L)	11.1 ± 4.1	11.2 ± 6.3	0.92			
Platelets (×10^9^/L)	233 ± 80	235 ± 68	0.92			
hs-cTnI (ng/L) *	47.0 (15.0–421.0)	70.0 (32.5–161.5)	0.21			
NT-proBNP (pg/mL) *	7056 (3320–20,855)	4550 (1788–22,129)	0.89			
C-reactive-protein (mg/L) *	45.3 (29.7–96.7)	44.8 (15.3–100.8)	0.86			
Heart rate (bpm)	90 ± 14	82 ± 15	0.17			
Systolic blood pressure (mmHg)	133 ± 34	132 ± 23	0.95			
Diastolic blood pressure (mmHg)	76 ± 18	74 ± 13	0.71			
SpO_2_ (%)	95 ± 3	95 ± 4	0.85			

HR = hazard ratio; BAV = ballon aortic valvuloplasty; CAD = coronary artery disease; TIA = transient ischemic attack; COPD = chronic obstructive pulmonary disease; hs-cTnI = high sensitivity-cardiac troponin I; NT-proBNP = N-Terminal Pro-B-Type Natriuretic Peptide; and SpO_2_ = peripheral capillary oxygen saturation. * Median (interquartile range). Bold results are statistically significant.

**Table 5:** Univariable and multivariable analyses evaluating all-cause mortality at 180-day follow-up.

	DEAD (*n* = 20)	ALIVE (*n* = 26)	*p* Univariable	HR	95% C.I.	*p* Multivariable
Pre-operative BAV (%)	2 (10)	10 (37.0)	0.063	**0.13**	**0.03–0.63**	**0.011**
Age (years)	88.4 ± 4.6	86.5 ± 7.3	0.33			
Male sex (%)	7 (35.0)	4 (15.4)	0.067	**2.75**	**1.04–7.27**	**0.041**
Hypertension (%)	20 (100.0)	26 (100.0)	1.00			
Diabetes mellitus (%)	2 (10)	8 (30.8)	0.12			
Dyslipidaemia (%)	6 (30.0)	16 (61.5)	0.038			
Heart failure (%)	16 (80.0)	16 (61.5)	0.17			
Known CAD (%)	7 (35.0)	9 (34.6)	0.91			
Atrial fibrillation (%)	7 (35.0)	6 (23.1)	0.19			
Previous TIA/stroke (%)	4 (20.0)	1 (3.8)	0.021	**9.10**	**2.27–36.4**	**0.002**
Chronic Kidney disease (%)	6 (30.0)	7 (26.9)	0.95			
COPD (%)	6 (30.0)	8 (30.8)	0.85			
Preexisting cognitive impairment (%)	3 (15.0)	4 (15.4)	0.84			
Charlson comorbidity index	5.6 ± 1.4	6.0 ± 2.3	0.94			
*Laboratory findings*						
Haemoglobin (g/dL)	10.8 ± 1.9	10.9 ± 1.4	0.88			
Creatinine (mg/dL)	1.2 ± 0.7	1.3 ± 1.4	0.76			
Serum glucose (mg/dL)	135 ± 46	132 ± 60	0.96			
Fibrinogen (mg/dL)	444 ± 153	422 ± 125	0.63			
White blood cells (×10^9^/L)	13.1 ± 7.7	9.7 ± 3.4	0.062			
Platelets (×10^9^/L)	243 ± 84	229 ± 57	0.58			
hs-cTnI (ng/L) *	64.5 (30.7–176.7)	49.5 (26.0–163.7)	0.19			
NT-proBNP (pg/mL) *	11,080 (3646–33,220)	3368 (1269–10,054)	0.27			
C-reactive-protein (mg/L) *	45.3 (18.6–75.6)	44.8 (13.6–101.5)	0.97			
Heart rate (bpm)	87 ± 14	81 ± 15	0.21			
Systolic blood pressure (mmHg)	130 ± 31	133 ± 20	0.56			
Diastolic blood pressure (mmHg)	75 ± 17	74 ± 12	0.84			
SpO_2_ (%)	95 ± 4	95 ± 4	0.55			

HR = hazard ratio; BAV = ballon aortic valvuloplasty; CAD = coronary artery disease; TIA = transient ischemic attack; COPD = chronic obstructive pulmonary disease; hs-cTnI = high sensitivity-cardiac troponin I; NT-proBNP = N-Terminal Pro-B-Type Natriuretic Peptide; and SpO_2_ = peripheral capillary oxygen saturation. * Median (interquartile range). Bold results are statistically significant.

## Data Availability

The data underlying this article will be shared on reasonable request to the corresponding author.
